# Health Dangers in Unemployment

**Published:** 1921-03-26

**Authors:** 


### Health Dangers in Unemployment.

The unemployed are not parading the streets with bands at their heads now, but they are there, and the problem they represent looks out of the eyes of every hungry man and half-fed child. To the cynical outsider it seems a matter almost for laughter that a society so highly organised as ours cannot find a way to carry on its existence without the shame of compulsorily idle men, but to those who realised in the war the terribly low physical standard of many of our industrial workers and the need when peace came of bending every effort to increase the numbers of A1 men and to diminish the number of C 3 it is pure tragedy, not because it means individual suffering, but because it means national loss. A plough left in the open all winter needs time and money before it can be got bright and ready again for work in the spring, and the human worker who has been long enough out of employment to exhaust his savings has to meet the full strain of work with a body enfeebled by deprivation. It is such subordinate facts as these which are often lost sight of, and yet they constitute a most serious part of the problem. Society gets a man back to work and heaves a sigh of relief. But even if he does his work adequately and without waste, he is running all the time grave physical risk of strain or illness which, though hidden at the moment, will crop up again in later years. In spite of the alarming amount of unemployment at present existing, there are probably fewer people actually starving now than in any other period when unemployment has been at all widespread. But the real danger to the community is not the few people who starve to death, but the great majority who go through a period of under-nourishment or malnutrition. Starvation ends in death, but in a period of under-nourishment the seeds are sown of weakness and disease which are never traced to their source. There are now many thousands of women unemployed, and a large proportion of these receive the State dole. It might almost be argued that a more efficient way of dealing with their cases would be to refuse them maintenance and make society face the consequences. Many of these women have enough to keep them alive on, but they may continue for months in a state of chronic semi-hunger. They will appear perfectly respectable. No one will think of them as "starving," but they will be eating unsuitable food and thus weakening their stamina and diminishing their capacity for effective motherhood. The effects of good feeding on the women of the country during the past few years are patent in the birth records. The effects of under-feeding and ill-feeding will in time be apparent too, not so quickly, nor so obviously, for they will take longer to work out, but they will be there, a serious debit item in the nation's balance-sheet of health. It should be the duty of the medical profession to bring to light these secondary effects of unemployment. There are more than have been mentioned above, and the nation should be taught to know them. All is not well when work has been found for the out-of-work. From the doctor's point of view the danger is often then only beginning. The hidden dangers of unemployment are indeed probably worse in the aggregate than those which are patent to all.

